# A novel upper tract ureteroscopic biopsy technique: the “form tackle”

**DOI:** 10.1590/S1677-5538.IBJU.2021.0499

**Published:** 2021-09-10

**Authors:** Dane E. Klett, Manaf Alom, Kevin Wymer, Aaron Potretzke

**Affiliations:** 1 Mayo Clinic Department of Urology Rochester MN USA Department of Urology, Mayo Clinic, Rochester, MN, USA

## Abstract

**Introduction and Objective:**

Upper tract urothelial carcinoma (UTUC) represents 5% of all urothelial malignancies ( 1 – 3 ). Accurate pathologic diagnosis is key and may direct treatment decisions. Current ureteroscopic biopsy techniques include cold-cup, backloaded cold-cup and stone basket ( 4 – 6 ). The study objective was to compare a standard cold-cup biopsy technique to a novel cold-cup biopsy technique and evaluate histopathologic results.

**Materials and Methods:**

We developed a novel UTUC biopsy technique termed the “form tackle” biopsy. Ureteroscope is passed into ureter/renal collecting system. Cold-cup forceps are opened and pressed into the lesion base (to engage the urothelial wall/submucosal tissue) then closed. Ureteroscope/forceps are advanced forward 3-10mm and then extracted from the patient. We compared standard versus novel upper tract biopsy techniques in a series of patients with lesions ≥1cm. In each procedure, two standard and two novel biopsies were obtained from the same lesion. The primary study aim was diagnosis of malignancy. IRB approved: 21-006907.

**Results:**

Fourteen procedures performed on 12 patients between June 2020 and March 2021. Twenty-eight specimens sent (14 standard, 14 novel) (Two biopsies per specimen). Ten procedures with concordant pathology. In 4 procedures the novel biopsy technique resulted in a diagnosis of UTUC (2 high-grade, 2 low-grade) in the setting of a benign standard biopsy. Significant difference in pathologic diagnoses was detected between standard and novel upper tract biopsy techniques (p=0.008).

**Conclusions:**

The “form tackle” upper tract ureteroscopic biopsy technique provides higher tissue yield which may increase diagnostic accuracy. Further study on additional patients required. Early results are encouraging.

**Figure:** Available at:http://www.intbrazjurol.com.br/video-section/20210499_Klett_et_al
